# A logistic regression analysis comparing minimalistic approach and intubation anaesthesia in patients undergoing transfemoral transcatheter aortic valve replacement

**DOI:** 10.1371/journal.pone.0227345

**Published:** 2020-02-05

**Authors:** Alexander Maier, Benedikt Hammerich, Frank Humburger, Thomas Brieschal, Timo Heidt, Wolfgang Bothe, Holger Schröfel, Klaus Kaier, Manfred Zehender, Jochen Reinöhl, Christoph Bode, Constantin von zur Mühlen, Peter Stachon

**Affiliations:** 1 Department of Cardiology and Angiology I, Faculty of Medicine, Heart Centre Freiburg University, University of Freiburg, Freiburg, Germany; 2 Department of Anaesthesiology and Critical Care, University Hospital Freiburg, Faculty of Medicine, University of Freiburg, Freiburg, Germany; 3 Department of Cardiovascular Surgery, Heart Centre Freiburg University, Faculty of Medicine, University of Freiburg, Freiburg, Germany; 4 Faculty of Medicine and Medical Centre-University of Freiburg, Institute of Medical Biometry and Statistics, Freiburg, Germany; 5 Edwards LifeScience, Nyon, Switzerland; Klinikum Region Hannover GmbH, GERMANY

## Abstract

**Aims:**

Patients with postoperative delirium (POD) after transcatheter aortic valve replacement (TAVR) are ventilated and hospitalized longer and suffer increased in-hospital mortality. This study hypothesized that a minimalistic approach with conscious sedation during transfemoral aortic valve replacement (TF-AVR) protects against delirium, time of mechanical ventilation, and increased length of stay in intensive care unit (ICU) compared to intubation anaesthesia.

**Methods and results:**

308 patients which underwent TF-AVR in our centre between 01/2013 and 08/2017 were retrospectively evaluated regarding postoperative delirium, time of mechanical ventilation, and days in ICU. TF-AVR was performed with intubation anaesthesia in 245 patients and with conscious sedation in 63. The operative risk estimated by the logEUROScore was similar in both groups (intubation: 13.28 +/-9.06%, conscious sedation: 12.24 +/-6.77%, p = 0.395). In the conscious sedation group procedure duration was shorter (0.61 +/-0.91h vs. 1.75 +/-0.96h, p<0.001). The risk for intraprocedural complications was not influenced by the anaesthesia method (OR conscious sedation instead of intubation 1.66, p = 0.117), but days on ICU (-2.21 days, p<0.0001) and minutes of mechanical ventilation (-531.2 min, p < 0.0001) were reduced. Furthermore, the risk of POD was decreased when TF-AVR was performed under conscious sedation (6.35% vs. 18.18%, OR 0.29, p = 0.021).

**Conclusions:**

Time of mechanical ventilation, risk of POD, and days on ICU were substantially reduced in patients who underwent TF-AVR under conscious sedation. Our data suggest that TF-AVR with conscious sedation is safe with a beneficial postoperative course in clinical practice, and should be considered the favoured approach.

## Introduction

Transcatheter aortic valve replacement (TAVR) is the standard care for patients with high-grade aortic valve stenosis at increased operative risk. Studies with patients at low-risk are in progress [1–5]. Consequently, the number of patients undergoing TAVR constantly rises [6, 7]. Patients with severe aortic valve stenosis often are at high risk for postoperative delirium (POD) due to pre-existing conditions such as dementia, advanced age, heart failure, or atrial fibrillation [8].

POD is defined as a potentially lethal state caused by acute or subacute brain failure with disturbance of consciousness, hyper- or hypoactivity, and disorientation or perceptual disturbance. It may trigger cognitive decline and lasting dementia [9–11]. For cardiac surgery it was shown that POD is a predictor for worse postoperative course [12]. The incidence of a relevant POD after TAVR was 8% in Germany in 2014, and higher after non-TF than TF procedures [13]. Male sex, higher NYHA class, and atrial fibrillation are risk factors for POD [8]. Patients with POD after TAVR were ventilated and hospitalized longer and suffered an increased risk for in-hospital mortality [8].

Unfortunately, common therapeutic strategies for the treatment of delirium are ineffective and time consuming, with prolonged need for ICU stay and a subsequent high expenditure of health care resources [14, 15]. Hence prevention of POD is the best treatment [16].

In recent years, a minimalistic approach for TF-AVR under conscious sedation without the need for intubation and mechanical ventilation during the procedure has been established [17, 18]. Thus, the use of anaesthetics and opioids can be reduced, resulting in faster patients’ reorientation without development of relevant POD. Therefore, we hypothesized that a minimalistic approach with conscious sedation may reduce time of mechanical ventilation, POD, and days on ICU after TF-AVR in comparison to intubation anaesthesia.

To test this hypothesis, we retrospectively analysed data of patients undergoing TF-AVR at our centre in either conscious sedation or intubation anaesthesia, considering time of mechanical ventilation, POD, and days on ICU.

## Materials and methods

### Study design

In this observational retrospective cohort study, we included 362 consecutive patients which underwent TF-AVR under intubation anaesthesia or conscious sedation in the Department of Cardiology and Angiology I of the Heart Centre Freiburg University between January 2013 and August 2017. An interdisciplinary heart team determined the treatment strategy and anaesthesia. The local ethics committee of the University of Freiburg approved the retrospective collection of data in the field of TAVR in our hospital (No. 29/11), and the study complies with the Declaration of Helsinki. Informed consent was waived by the ethics committee. Patient data were not anonymized before release to us, but informed consent was not required by the ethics committee.

### Data acquisition

All data was collected in a registry using the hospital documentation system (MeDoc, Freiburg, Germany). Baseline characteristics and procedural data were collected retrospectively and transferred to Microsoft Excel (Redmond, USA).

Previous dementia and previous delirium were assessed using all available previous physician letters from our hospital and other hospitals as well as primary care physicians.

Intraprocedural complications were defined in accordance with the updated standardized endpoint definitions for transcatheter aortic valve implantation of the valve academic research consortium (VARC-2) [19] as complications occurring either at the site of vascular access (major and minor bleeding, femoral artery stenosis, femoral artery occlusion, femoral artery or aortic dissection, fistula, aneurysm, and unspecific minor complications) and / or at the valvular region (pericardial effusion, dislocation of the prosthesis, annular rupture, conversion to surgery, bradycardia, ventricular fibrillation, and unspecific minor complications). Procedure time was defined as “first to last stitch”. Applied volume of contrast agent was measured in millilitres during the TF-AVR procedure. Rapid pacing was counted manually. Time of mechanical ventilation and duration of the TAVR procedure were measured in minutes using our ICU documentation system (COPRA, COPRA System GmbH, Germany). Duration of ICU stay was measured in days.

Diagnosis of POD was made by experienced ICU physicians of our department supported by the”Delirium Detection Score”(DDS) and „Nursing Delirium Screening Scale”(NU-DESC) [20, 21]. Delirium was defined as > 7 points in DDS and ≥ 2 points in NU-DESC. Occurrence of delirium was defined as occurrence of delirium during patients’ stay on the ICU. Patients’ neurological status was evaluated at least three times per day by experienced physicians. Neurological assessment on the general ward was performed at least once a day by an experienced physician during the daily visitation. Once delirium occurred on the general ward, patients were transferred back on the ICU.

### TF-AVR procedure

After heart team decision, patients were assigned for TAVR either with conscious sedation or intubation anaesthesia. Conscious sedation was generally established in 2016; afterwards only patients with contraindication for conscious sedation were assigned for general anesthesia. A frailty assessment was not performed. Intubation anaesthesia was performed using opioids, propofol, sevoflurane, or benzodiazepines at the anaesthesiologist’s discretion. All patients under conscious sedation received intravenous dexmedetomidine only, combined with local anaesthesia with Xylocaine. TF-AVR procedure was performed as described elsewhere [2].

### Statistical analysis

Differences in baseline characteristics and procedure times were calculated using the Student’s t-test and the chi-square test for continuous and categorical variables, respectively. The impact of anaesthesia method on postoperative delirium, time of mechanical ventilation, and days in ICU was determined using logistic or linear regression analyses. In the latter case, standard errors were calculated using robust variance estimation to account for misspecifications of the models regarding the distribution time of mechanical ventilation and days in ICU. In order to account for possible differences in the pre-procedural risk of patients undergoing conscious sedation or intubation anaesthesia, all regression analyses were risk-adjusted using the logistic EuroSCORE. All analyses were carried out using Stata 15 (Stata Corp, College Station, Texas).

## Results

### Baseline characteristics

**Fig 1:**
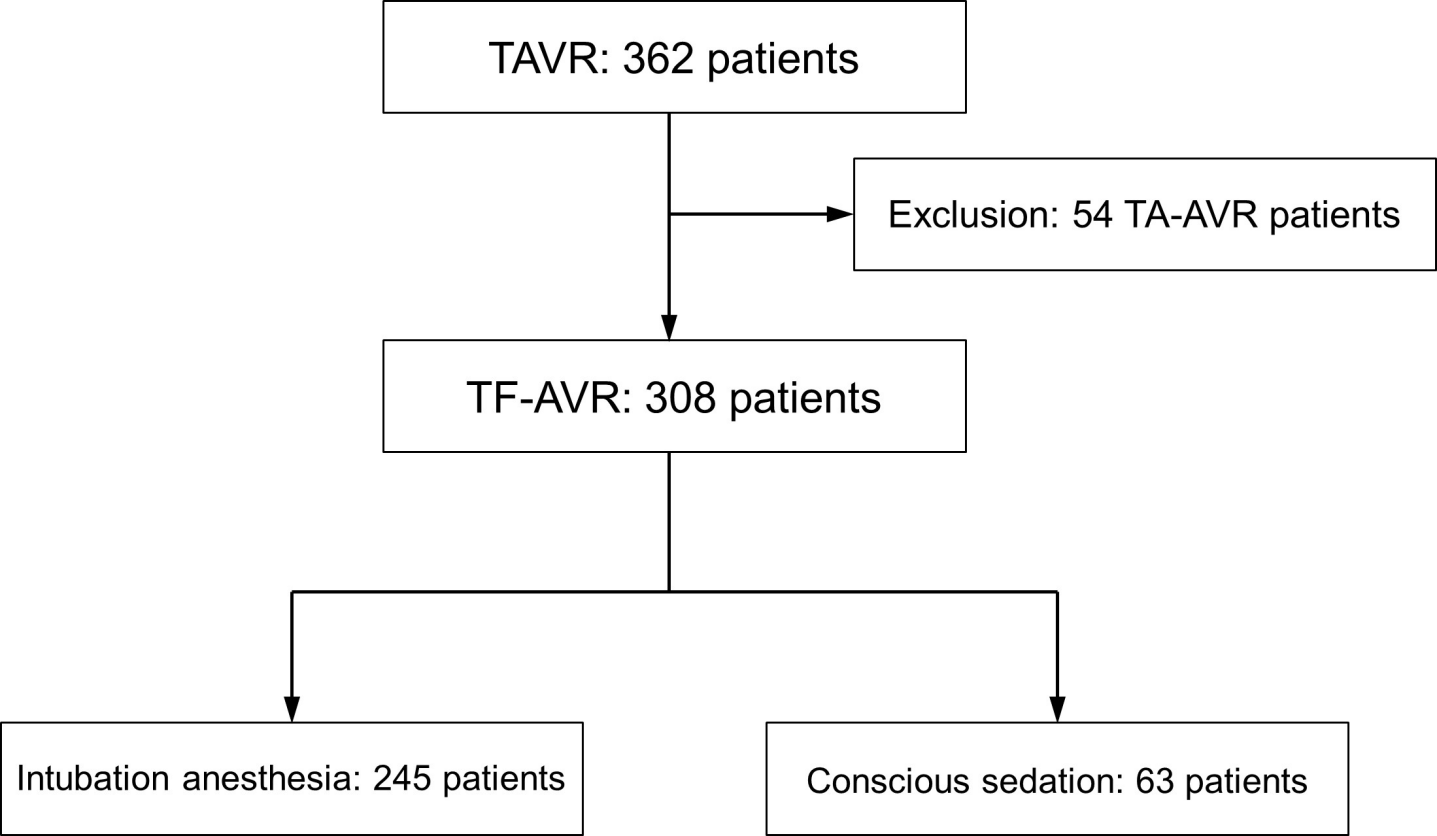
Study design.

Between January 2013 and August 2017, 362 patients underwent TAVR in the Department of Cardiology and Angiology I of Heart Centre Freiburg University. We excluded 54 patients which received TA-AVR. 308 patients received TF-AVR and were assessed for this study. 63 received TF-AVR with conscious sedation and 245 with intubation anaesthesia (**Fig 1**). Patients were of similar age (conscious sedation: 81.38 +/- 6.54, intubation anaesthesia: 81.15 +/- 6.45, p = 0.801) and similar logEUROScore (conscious sedation: 12.24 +/- 6.77%, intubation anaesthesia: 13.28 +/- 9.06%, p = 0.395). Previous dementia was present in 2% of the conscious sedation group and 4% of the intubated group (p = 0.341). NYHA classification (conscious sedation: 2.58 +/- 1.00, intubation anaesthesia: 2.81 +/- 0.87, p = 0.073), left ventricular ejection fraction (EF, conscious sedation: 50.61 +/- 9.97%, intubation anaesthesia: 49.44 +/- 10.79%, p = 0.446), and mean trans-aortic gradient (conscious sedation: 42.43 +/- 14.33 mmHg, intubation anaesthesia: 41.59 +/- 14.19 mmHg, p = 0.684) were similar in both groups. The rate of patients with diabetes mellitus was 27% in the conscious sedation group and 34% in the intubation anaesthesia group (p = 0.297). Smoking (25% vs. 16%, p = 0.080) and atrial fibrillation (47% vs. 42%, p = 0.501) occurred more often in the conscious sedation group. No differences were found in gender distribution, previous rate of stroke, previous delirium, previous atherosclerotic diseases, and previous hypertension (see **Table 1**).

**Table 1:** Baseline characteristics.

	Intubation anesthesia n = 245		Conscious sedation n = 63	
	mean / median	SD / IQR	mean / median	SD / IQR
Age	81.15	6.45	81.38	6.54
Female	54%		54%	
Ejection fraction	49.44	10.79	50.61	9.97
Mean trans-aortic gradient	41.59	14.19	42.43	14.33
NYHA	3	2–3	3	2–3
Previous dementia	4%		2%	
Previous stroke	13%		13%	
Previous delirium	1%		2%	
Atherosclerotic disease	23%		22%	
Atrial fibrillation	42%		47%	
Arterial hypertension	84%		81%	
Diabetes	34%		27%	
Smoking	16%		25%	
logEuroSCORE (isolated AVR)	13.28%	9.06%	12.24%	6.77%

### Intraprocedural complication rate

Complications at the site of vessel access were major and minor bleeding, femoral artery stenosis, femoral artery occlusion, femoral artery or aortic dissection, fistula, aneurysm, and unspecific minor complications. Complications in the valvular region were pericardial effusion, dislocation of the prosthesis, annular rupture, conversion to surgery, bradycardia, ventricular fibrillation, and unspecific minor complications. We found no significantly higher risk for intraprocedural complications in either of the groups (OR = 1.63, 95% CI 0.88–3.01, p = 0.117). Post dilatation was observed among patients undergoing intubation anesthesia only (5 cases, 2.04%, 0 cases among conscious sedation, p = 0.587). The same is true for dislocation where 4 cases (1.63%) were observed for intubation anesthesia and 0 cases among patients undergoing conscious sedation (p = 0.585).

The length of the procedure was reduced with conscious sedation, with a mean procedure time of 0.61 +/- 0.91 h, whereas the procedure time with intubation was 1.75 +/- 0.96 h (p < 0.001).

The volume of injected contrast agent was lower in the conscious sedation group (235.45 +/- 94.38 ml vs. 267.48 +/- 141.22 ml, p = 0.097). Rapid pacing was performed fewer times under conscious sedation (0.88 +/- 0.59 vs. 1.34 +/- 0.77, p < 0.001). (**Table 2)**.

**Table 2:** Procedural data. A: length of procedure, contrast agent in ml and numbers of rapid pacing. B: Odds ratio for intraprocedural complications.

**A**		**Intubation anaesthesia**		**Conscious sedation**	
		mean	SD	mean	SD
	Length of procedure [h]	1.75	0.96	0.61	0.91
	Contrast agent [ml]	267.48	141.22	235.45	94.38
	n rapid pacing	1.34	0.77	0.86	0.59
**B**	**Odds Ratio for intraprocedural complications**	Odds ratio	p-Value	[95% CI]	
	Local anaesthesia instead of intubation	1.63	0.12	0.88	3.01

### Days on ICU with conscious sedation or intubation anaesthesia after TF-AVR

**Fig 2:**
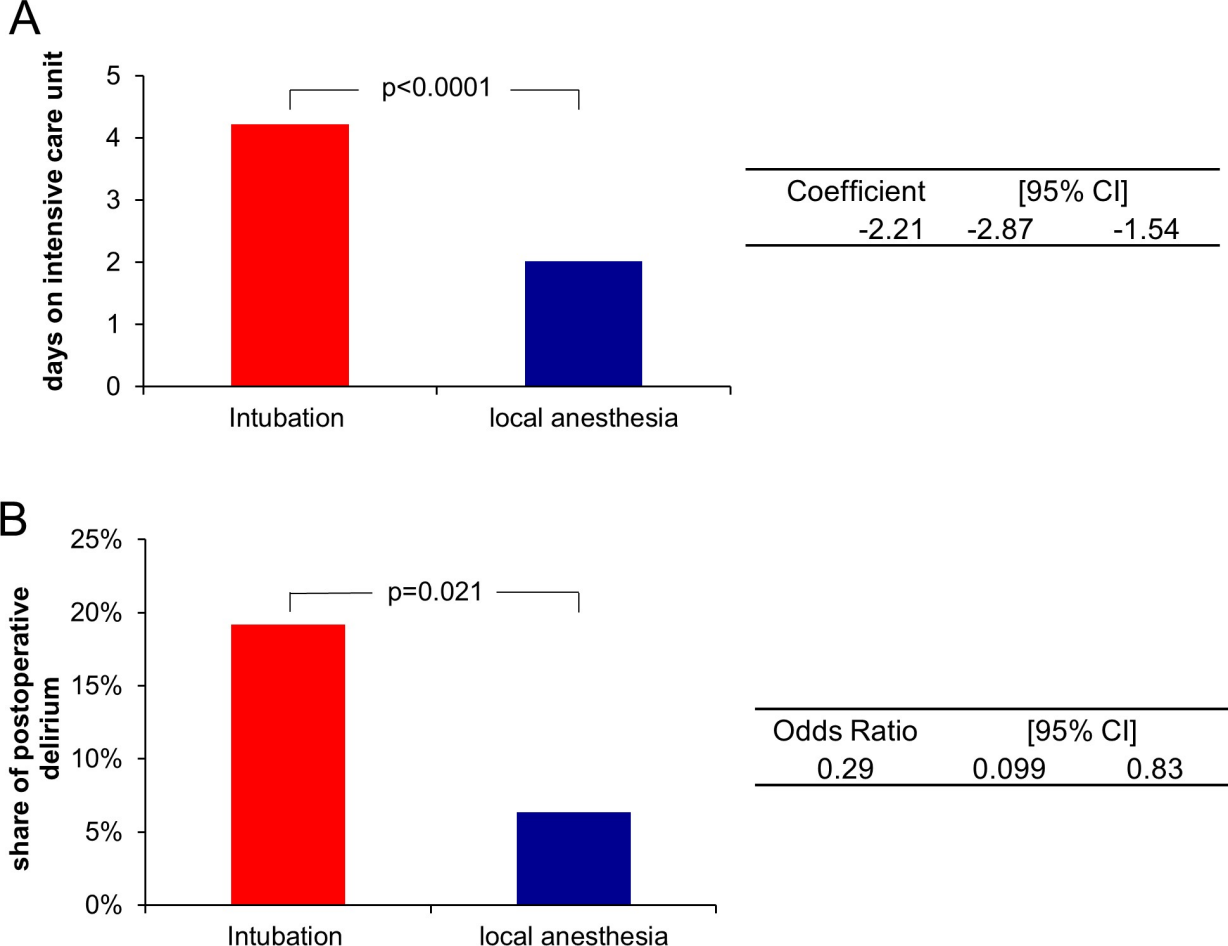
Days on intensive care unit. (A). Share of postoperative delirium (B).

After TF-AVR under conscious sedation, mean ICU stay duration was 2.02 days, whereas mean duration of ICU stay after TF-AVR with intubation anaesthesia was 4.22 days. The first and last day on ICU are counted as full days. The difference between the two groups was 2.21 days (p < 0.001, 95% CI -2.87–1.54, see **Fig 2A**). After risk-adjustment using the logistic EuroSCORE, conscious sedation was still associated with reduced ICU stay (-2.21 days, p < 0,001, 95% CI -2.89 - -1.53).

### Time of mechanical ventilation with conscious sedation or intubation anaesthesia

Mean time of mechanical ventilation after TF-AVR under conscious sedation was 4.29 min, whereas mean ventilation time after TF-AVR with intubation anaesthesia was 529.03 min. The reduction in the duration of mechanical ventilation was 531.2 min (p < 0.001, 95% CI -791.8 - -270.6). On average more than 99% of ventilation time could be avoided through TF-AVR under conscious sedation independently of the logEUROScore. After risk-adjustment using the logistic EuroSCORE, conscious sedation was still associated with reduced time of mechanical ventilation (-524.8 min, p < 0,001, 95% CI -779.5 - -270.1).

### Risk for development of POD

6.35% of patients which received TF-AVR under conscious sedation developed a relevant POD, whereas 19.18% of the patients which received TF-AVR under general anaesthesia did. As a result, the risk of POD after conscious sedation is significantly reduced in comparison to TF-AVR under intubation anaesthesia (odds ratio 0.29, p = 0.021, 95% CI 0.099–0.83, **Fig 2B**). After risk-adjustment using the logistic EuroSCORE, the decreased risk for development of POD for conscious sedation remained identical, and conscious sedation was still associated with reduced time of mechanical ventilation.

### POD and risk for new need of pacemaker

44.44% (20 patients) of the patients with POD needed a new pacemaker, whereas only 26.22% (59 patients) of patients without POD needed a new pacemaker (odds ratio 2.25, p = 0.015, 95% CI 1.163–4.345). After risk-adjustment using the logistic EuroSCORE, the decreased risk for new need of a pacemaker in patients without POD remained identical. In contrast, 11.76% (6 patients) of the patients with pre-existing pacemaker developed a relevant delirium and 12.45% (32 patients) of patients without pre-existing pacemaker had a relevant delirium (odds ratio 0.92, p = 0.875, 95% CI 0.365–2.361).

## Discussion and limitations

The present study demonstrates that TF-AVR under conscious sedation protects against prolonged mechanical ventilation, prolonged ICU stay, and POD compared to TF-AVR under intubation anaesthesia. Furthermore, TF-AVR under conscious sedation did not influence the safety of the TF-AVR procedure.

Baseline characteristics were similar between patients undergoing TF-AVR under conscious sedation or intubation anaesthesia. The overall risk profile was similar to other TAVR studies [2, 4, 13]. TF-AVR under conscious sedation could be expected to increase operative complications, since patients may be more agitated and transoesophageal echocardiography is missing. However, in line with previous studies, we did not find an increase in peri-interventional complications such as in-hospital mortality, stroke/transient ischemic attack, and major bleeding [22]. Even the procedure time was shorter. This fact however is more likely due to the increasing experience over the time than due to the local anaesthesia. However, it shows that local anaesthesia does not seem to increase complexity of the intervention. The interdisciplinary heart team, which includes an anaesthesiologist, decided for each patient individually whether conscious sedation was possible.

Time on ICU and time on mechanical ventilation were markedly reduced. The time on mechanical ventilation on ICU after intubation anaesthesia was 529 min. In contrast, unplanned intubation was a rare condition and resulted in an average ventilation time of only four minutes in patients undergoing TF-AVR under conscious sedation. The entire ventilation time in the conscious sedation group was indeed due to a single patient who underwent unplanned intubation. No other patients in this group had to be ventilated.

Time on ICU was reduced by around two days after TF-AVR under conscious sedation. This reduction was caused by several factors. First, mechanical ventilation was rarely necessary, since need for unplanned intubation rarely arose. Thus, ventilation-associated complications did not occur. Second, the incidence of POD was reduced. Third, only dexmedetomidine was used in patients with conscious sedation, which is known to improve postoperative awakening with reduced risk for POD [3, 23]. Furthermore, the combination of anaesthetics used for intubation may influence cardiovascular circulation, resulting in a higher need for catecholamine and therefore prolonged stay on ICU. Fourth,—as an important confounder—conscious sedation was established in 2016, so technical developments and increased interventional experience also reduced time on ICU, in addition to the effects of the conscious sedation. Nevertheless, using a minimalistic approach only a minimum time of ICU treatment is necessary to monitor conduction disturbances, acute access site complications or other severe cardiac complications, if patients do not suffer from any other condition that makes ICU treatment necessary, e.g. serious infection or acute decompensated heart failure.

An important finding of the present study was the significant reduction of POD, with an adjusted OR of 0.29 favouring conscious sedation, since prevention of POD is more effective than treatment after occurrence [15]. The rate of POD under general anaesthesia is similar to that in a former study with a comparable methodology of POD measurement [24].

Reasons for significantly fewer PODs after TF-AVR under conscious sedation could be that no reorientation is needed without awakening after general anaesthesia, and the use of dexmedetomidine [23, 25], which is used in treatment of POD on ICU. The combination of different anaesthetics and opioids is known to increase the risk for a POD. Another important factor which could protect against POD is that no weaning from assisted ventilation is needed. With conscious sedation, analgesia can be controlled more easily because patients are able to call for help if any problem that could lead to POD occurs, which is not possible under general anaesthesia. Hemodynamics are also more stable under conscious sedation, and less need for opioids is described [17, 18]. Additionally, the shorter procedure and faster mobilization after TF-AVR under conscious sedation might also have a positive impact [8].

We found an association between POD and new need for pacemaker after TAVI, but no protective effect of a pre-existing pacemaker. Reasons for the higher risk for a new pacemaker in POD patients could be a prolonged hospitalization because of conduction disturbances and the implantation of the new pacemaker with a second sedation. A pre-existing pacemaker did not protect against POD in our analysis.

POD after TF-AVR prolongs hospital stay by 6 days [8]. Thus, conscious sedation saves health care resources through reduction of POD, of expensive ICU stays, and of the length of overall hospital stay. This study demonstrates the evolution of a successful treatment methodology, in this case TAVR, in an exemplary way. It can be seen in line with other studies finding less invasive TAVR methodologies resulting in less POD, in particular for TF-AVR in comparison to TA-AVR [25–27]. Conscious sedation makes the procedure itself faster, the postoperative period safer, and the hospital stay more efficient. This is of advantage for patients, health care professionals, and health care systems.

This study has several limitations. First, it is a retrospective single centre study, with the known limitations of that study type. Furthermore, conscious sedation has become the standard anaesthesia in our centre since the beginning of 2016 and we included patients retrospectively from 2013. Thus, the intubation anaesthesia group is a historic control and outcomes may have also improved due to learning curve effects. However, the data of our study are of good quality: although the patient number of the conscious sedation group is lower than in the general anaesthesia group, baseline characteristics are balanced and both groups are of sufficient size. Proof of reduction of POD and ICU stay could be provided by a randomized multicentre trial. In this case, blinding would probably be difficult because the methodology of sedation is obvious for all involved.

High contrast agent volumes can potentially influence POD. However, the contrast agent volume was comparable in both groups, and our data is in line with previous studies. Previous studies showed a shorter length of stay and lower in-hospital and 30-day mortality in TAVR for conscious sedation compared to general anesthesia [28]. Advantages of reduced procedural time, faster recovery and reduced cost were also described before [29]. All these facts let us expect a lower delirium rate and shorter ICU stay duration. Our data confirmed these expectations in a single center study.

## Conclusions

In conclusion, conscious sedation after TF-AVR is safe and protects against POD, mechanical ventilation, and prolonged ICU stay in comparison to general anaesthesia. The data of our retrospective study suggest that TF-AVR with conscious sedation is safe with a beneficial postoperative course in clinical practice, and should be the favoured approach.

### Impact on daily practice

Minimalistic approach using conscious sedation in patients undergoing TF-TAVR is safe, reduces the risk for prolonged ventilation, stay on ICU, and the risk of developing a delirium. Therefore, it should be considered the favored approach.

## Supporting information

S1 Data (XLSX)
